# Design, Implementation, and Evaluation of Self-Describing Diabetes Medical Records: A Pilot Study

**DOI:** 10.2196/medinform.6862

**Published:** 2017-05-02

**Authors:** Azadeh Kamel Ghalibaf, Zahra Mazloum Khorasani, Mahdi Gholian-Aval, Hamed Tabesh, Mahmood Tara

**Affiliations:** ^1^ School of Medicine Department of Medical Informatics Mashhad University of Medical Sciences Mashhad Islamic Republic Of Iran; ^2^ School of Medicine Endocrine Research Center Mashhad University of Medical Sciences Mashhad Islamic Republic Of Iran; ^3^ School of Health Department of Health Education and Health Promotion Mashhad University of Medical Sciences Mashhad Islamic Republic Of Iran

**Keywords:** diabetes electronic medical record, information tailoring, patient education, technology acceptance model

## Abstract

**Background:**

Each patient’s medical record consists of data specific to that patient and is therefore an appropriate source to adapt educational information content.

**Objectives:**

This study aimed to design and implement an information provision system based on the medical records of diabetic patients and to investigate the attitudes of users toward using this product.

**Methods:**

The study was organized into three phases: need analysis, design and implementation, and final evaluation. The aim of the need analysis phase was to investigate the questioning behavior of the patient in the real-world context. The design and implementation phase consisted of four stages: determining the minimum dataset for diabetes medical records, collecting and validating content, designing and implementing a diabetes electronic medical record system, and data entry. Evaluating the final system was done based on the constructs of the technology acceptance model in the two dimensions of perceived usefulness and perceived ease of use. A semistructured interview was used for this purpose.

**Results:**

Three main categories were extracted for the patient’s perceived usefulness of the system: raising the self-awareness and knowledge of patients, improving their self-care, and improving doctor-patient interaction. Both patients and physicians perceived the personalized sense of information as a unique feature of the application and believed that this feature could have a positive effect on the patient’s motivation for learning and using information in practice. Specialists believed that providing personal feedback on the patient’s lab test results along with general explanations encourages the patients to read the content more precisely. Moreover, accessing medical records and helpful notes was a new and useful experience for the patients.

**Conclusions:**

One of the key perceived benefits of providing tailored information in the context of medical records was raising patient awareness and knowledge. The results obtained from field observations and interviews have shown that patients were ready to accept the system and had a positive attitude when it was put into practice. The findings related to user attitude can be used as a guideline to design the next phase of the research (ie, investigation of system effectiveness on patient outcomes).

## Introduction

Diabetes is a chronic metabolic disease that is highly prevalent in Iran as well as the entire world. Type-2 diabetes is among the most common chronic illnesses in Iran, with a prevalence rate greater than 14% for the population aged 30 years and older [1]. There is no cure for this disease and it requires continuous lifelong care. Results from previous research have shown that raising the knowledge of diabetic patients and their awareness empowers them to better manage their disease [2,3].

Medical records are rich sources for the medical background of patients, and thus can be used as a basis for making medical decisions. In recent years, with the advent of electronic medical records (EMR), the issue of a patient accessing one’s medical record has gained more attention than ever before. According to the Health Insurance Portability and Accountability Act of 1996 (HIPAA), patients must be able to see a copy of their records [4]. The Institute of Medicine also advocates for unrestricted patient access to medical records. Existing literature suggests that providing patients access to their medical records can improve their knowledge, facilitate a more collaborative relationship between the provider and patient, patient adherence, greater patient involvement in self-care, and more satisfaction [5,6]. The key point is that the realization of the above-mentioned advantages concerning patient-accessible medical records is possible only when the record content is comprehensible to the patient. A relevant review article [5] has shown that patients commonly had difficulty understanding at least part of their records. Providing explanations about the record not only helps to improve the self-awareness of one’s disease status, but also enjoys an educational aspect to help promote patient knowledge and skills, especially if the information provided is structured and relevant to each patient’s medical conditions and needs. According to the elaboration likelihood model, when people perceive relevant information about their conditions, they become further motivated to read it and remember more details [7].

Information tailoring systems use an internal representation of user conditions and needs, which is referred to as a “user model” or “user profile.” A user model represents the system’s beliefs about the user. Hence, it may simply contain demographic information or sophisticated factors such as the state of the disease, user’s attitude, interest, preference, and knowledge [8]. Decisions on the number and types of factors involved in the user model depend on the desired dosage of tailoring and the purpose of the system.

Tailoring research began appearing in the literature in the 1990s, and with advances in computer technology, has increased dramatically in recent years [9]. On the basis of the researchers’ primary discipline and expertise, we have found two main approaches in this field. The approach led by the computer science community is focused on advanced and intelligent technological methods, but deals less with real-world issues such as implementation context, target user characteristics, and outcome evaluation. Such projects as LEAF (Layman Education and Activation Form) [10], HealthDoc [11], and Migraine [12] are examples of tailored document generation systems within this category.

The second approach relies greatly on evaluating the efficiency of tailored materials compared with generic ones, but generally uses simpler technological methods to generate automatically the tailored print-based materials. A common drawback of research done with this approach is insufficient reporting of tailoring system architecture, which is referred to as the “black box” [9]. We have attempted to fill the existing gap between these two approaches by describing the design and implementation of the system in more detail.

We used the medical record data of patients as the tailoring profile, in which the relevant information retrieved from the library of content is provided to the patient. The system was designed as stand-alone software that can be used in almost any health care environment regardless of the availability of the patient’s electronic record. In the presence of an electronic record, our system can be used as an add-on module to the existing EMR, automatically retrieving the patient’s data from the EMR. However, there might be a need for simple middleware to convert and adapt the recorded items in EMR to those defined in the user’s profile. Embedding the system in the existing EMR system can facilitate its acceptance and practicality.

Research has shown that the target audience’s acceptance of the system is a key factor in the success of an information system within a clinical setting. There are two groups of target users whose benefits should be taken into account: final users, who work directly with the system, and clinicians, who confirm and validate the system and support its usage in practice. User experience is a field of study that analyzes the user’s emotions, behaviors, and attitudes toward using a product, system, or service [13].

In recent years, different models and frameworks have been introduced to evaluate information systems [14,15]. Technology acceptance model (TAM) is considered the most influential and commonly employed theory for describing an individual’s acceptance of information systems. It has been adapted from the theory of reasoned action, which is based on the assumption that one’s intention to act out a certain behavior, such as using the system or reading the provided information, is predictable from one’s attitude toward that behavior [16]. TAM is the basis for evaluating our system.

Our aim was to design and implement a tailored information provision system based on diabetic patient medical records and to investigate user attitudes toward this product. The research plan was conducted in three phases: need analysis, design and implementation, and final evaluation of the system. First, we have described the methodology used in each phase of the study. Then we have reported the findings of each phase, and finally, we discussed the results and experiences gained along with the causes and factors involved.

## Methods

In this section, the methodology used in each phase is explained respectively.

### Phase I: Need Analysis

We used field observations of patients in order to identify and prioritize the information needs of the target population. Observation was done in two distinct settings: physicians’ visiting sessions for 22 hours, and diabetes educational classes for 10 hours. In the first setting, the researcher took a nonparticipant observer’s role in doctor-patient visits and only took field notes. In the second setting, the researcher adopted a patient’s role and participated in classes like other ordinary participants. She took notes of the patients’ questions and investigated intragroup behavior. We followed a two-step approach to analyze the noted data. We first assigned each question a label to indicate the main topic or concern, and then classified questions based on their labels. In the second step, we performed content analysis for each question to identify the underlying concerns. The findings have been reported in the results section.

### Phase II: Design and Implementation

The designing procedures of the tailored self-describing diabetes medical records were conducted in four stages as described in detail in this section.

#### Stage I: Determining the Minimum Dataset for Diabetes Medical Records

In order to identify a set of items usually recorded in the visits of diabetic patients, the sample paper-based records from five diabetes clinics in Mashhad city were gathered and analyzed. After *eliminating duplicated items,* a checklist was designed with five main categories: demographic information, symptoms, medical history, medication, and lab tests and measurements, with a number of subcategories for each. The checklist was provided to three subspecialists in endocrinology to determine the necessity of including each item. They could also add new items into the “others” section if needed. Items selected by the majority of specialists in the first round of Delphi, along with the newly added items, comprised a checklist for the second round of Delphi. Finally, 110 items were confirmed by the expert panel, including 10 items in the demographic category, 25 items in the symptoms category, 15 items in the medical history category, 45 items in the medication category, and 15 items in the lab test category, all of which included the minimum dataset for diabetes medical records.

#### Stage II: Content Collection and Validation

We initially needed a structure for collecting and presenting content that corresponded to items in each category. These structures were obtained based on the results of the need analysis phase. As an example, the structure of information for the items in the symptoms category includes four sections: symptom’s definition, diagnostic symptoms, causes of occurrence, and advice on prevention and treatment. The information content was collected from credible online and printed sources. Once the comprehensibility and simplification rules—such as lexical simplification, sentence shortening, bullet-points, and so on—were applied to the corpus of the text, we had to validate its quality and correctness. To do this, we provided a printed version of explanations based on the patient’s record with a quality assessment checklist to a small representative sample of clinicians and patients. Participants could also comment in free texts. Once the clinicians confirmed the information, it was stored in the system’s database.

#### Stage III: Design and Implementation of Diabetes EMR

The system works in two modes: doctor and patient. We used Microsoft Visual Studio and Microsoft Access database for development.

During each visit, the doctor-user should facilitate the data entry process by entering the patient’s data into the system by choosing from among the existing options in the program interface. The doctor can also observe the information about the patient’s previous visits by selecting the visit date from the presented list on the screen.

The patient-user can also log in to the system with his or her username and see the record content in two ways (screenshots are available in Multimedia Appendix 1): session-based and topic-based.

*Session-based* content shows a complete list of all visits based on the visit date. A click on each date reveals all data values recorded on that particular session in three distinct columns titled symptoms and diseases, medication, and lab tests. Each record item is in the form of hypertext; it is clickable and capable of providing explanations. *Topic-based* content provides a visual abstract overview of the chronological progress for the selected data elements in a patient’s record.

In our proposed EMR system there is a possibility of providing explanations for each item in the patient’s record. Clicking on a term opens up a window with explanations. The content of the explanation can be divided into two sections based on the degree of tailoring.

The first section is less tailored, which includes relevant general knowledge about the selected item, and is presented uniformly to all patients. Technically speaking, canned text is used to produce the content of this section, which is stored in advance in the system’s library of content. In case all conditions are met, this text is retrieved.

The second section is highly tailored, which interprets the patient’s medical condition based on the content of his or her record. To generate the text in this section, the shallow natural language generation approach is used with schema or fill-in-the-blanks strategy.

**Figure 1:**
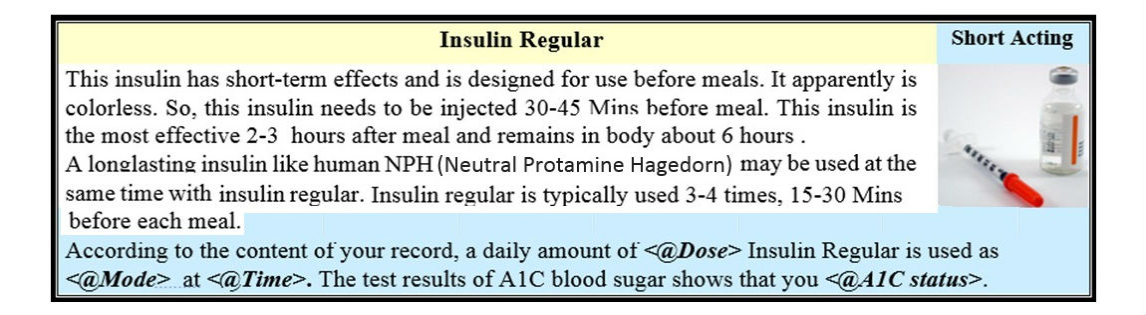
System explanations on insulin regular.

For example, once the patient clicks on “Insulin Regular,” at first a general explanation is provided about this type of insulin and its mechanism of action in the body. Then the second part is focused on a tailored explanation about how “Insulin Regular” should be administered by that specific patient in terms of dosage, timing, and so on (Figure 1; this page originally contained Persian text, shown here is a translation). This type of information provision is based on the assumption that integrating personal information from the patient’s medical record with general medical knowledge facilitates a better learning and higher self-awareness of the patient’s health status.

#### Stage IV: Data Injection Into the System

As there were no EMR in the clinic where we conducted the study, we had to manually enter data from patients’ paper-based records. To do this we used the clinic appointment-scheduling list to identify patients who would visit in the upcoming days. We then retrieved their medical records from the archive and manually entered the data into the system. On the basis of each patient’s record, a personalized document was produced by the system comprising the relevant information based on that patient’s medical status. The document was then printed and delivered to the patient in the form of a booklet in his or her upcoming visit session.

### Phase III: Evaluation

This phase deals with investigating the users’ attitude and perceived value of using the system in practice. The study was conducted in one of the leading health centers providing diabetes prevention, treatment, and management services in Mashhad city. The center was undergoing a switching transition from the traditional paper-based workflow to a digital format at the time of this study.

The evaluation was conducted with two groups of users: doctors and patients. We aimed to identify the key factors involved in the acceptance of the system by users. The methodology used was a semistructured interview based on the constructs of TAM. This model claims that one’s acceptance of an information system and intention to use it depends on the two constructs: perceived usefulness and perceived ease of use [17].

Perceived usefulness means one’s belief that using the system improves his or her performance. To assess this construct, interview questions were specified in advance in the interview guide based on Davis’s standard questionnaire that measures the scales defined in TAM [18] (a detailed description is provided in Multimedia Appendix 2). Interviewees were free to state their opinions about multiple aspects. During each interview session, the interviewer could ask more detailed and precise questions based on the respondent’s feedback. Sample questions are: “In your opinion, what advantages are involved in using this system?” “How does the system facilitate your needs?” or “In what ways can the use of this system be useful and helpful?”

Perceived ease of use refers to one’s expectation of the system’s simplicity in interactions, and identifying the influential factors. To assess this construct, we planned a two-stage approach: first, we introduced the system to the patient at the beginning of the interview session and showed how it worked in practice; then, we asked the patient to perform the task of entering his or her last visit data to experience how it feels to interact with the system. While working on the different parts of the system, the patient thinks aloud about his or her comments on the program’s facilities and interface design.

At the end of the session, we asked participants some open-ended questions in order to assess the user’s overall perception of the ease of using the program. The questions were derived from the standardized SUS (System Usability Scale) [19] and PUEU (Perceived Usefulness and Ease of Use) [18] and are available in Multimedia Appendix 2. The results of the data analysis are included in the following section.

## Results

In this section, the findings of the data analysis are reported for each phase of the study.

### Phase I: Need Analysis

Overall, 46 items were noted by the researcher. Once redundancies were removed, they were classified into nine categories: symptoms, oral drugs, insulin, lab tests, blood glucose test, diet, physical activity, psychological problems, and exposure to new circumstances (Multimedia Appendix 3). According to the purpose of this study, which was to provide explanations through patient records, only four of these thematic categories were found to be associated with the patient’s record: symptoms, oral drugs, insulin, and lab tests (Table 1).

Findings from this phase were then used to determine the structure of information in the design phase. The results from this phase revealed the structure of information to be provided for the patient based on their needs.

**Table 1:** Categorization of patients’ questions.

Topic-based category	Concept
Symptoms	Cause of the symptom
	Diagnostic symptoms
	Recommendations
Drugs	Side effects
	Dosage
	Administration and use
Lab tests	Definition
	Normal range
	Patient’s status

### Phase II: System Design and Implementation

In this research, content quality was controlled not only by health providers but also by patients.

#### Evaluating Content Quality by Clinicians

Before the system is available to patients, the quality of the content should be ensured. To do this, we asked three experts in the diabetes domain with over 10 years of experience (one endocrinology subspecialist, one general practitioner (GP) who had passed a diabetes course, and one nurse who was a diabetes educator) to evaluate content quality through a checklist (Multimedia Appendix 4). Four criteria were checked: accuracy, simplicity, usefulness, and adequacy. Each aspect is rated between 1 and 5. Table 2 provides mean scores and standard deviations for each aspect.

As it can be observed in Table 2, drugs and diseases received the lowest total scores. Experts believed that the content related to the drug’s mechanism of action is unnecessary and difficult for patients. A comparison of mean rating scores indicates that the nurse in charge of diabetes education rated the content quality lower than the physicians. One reason for this can be due to the nurse’s better knowledge of patients’ learning capabilities, which is a result of more time and closer contact the nurse spent with patients (Multimedia Appendix 4).

#### Evaluating Content Quality by Patients

Once the health providers approved the content, we inquired about patients’ opinions on the quality of information content. This time just the information items related to each patient’s record were available to five diabetic patients (three women and two men). The patients had two choices to access the information: on the computer screen or on paper. From the five patients, four requested the paper-based version. Therefore, the information related to every patient’s medical record was prepared in a booklet and provided for the patient in the visiting session along with the checklist. The patients had 1 month to read through the booklet and fill out the checklist. Each piece of information was rated on a Likert scale based on four criteria: comprehensibility, practicality, essentiality, and novelty. Moreover, patients were able to add their comments in the explanation section whenever needed.

The patients commented differently on content quality. One reason for such diversity can be due to the varying size and type of information provided for each patient, which itself is a result of the patients’ different conditions. The other reasons for this diversity maybe the patients’ differing literacy and knowledge levels. Overall, all the participants rated the content quality satisfactory. The patients’ rating of comprehensibility, essentiality, and novelty was good or very good.

### Phase III: Final System Evaluation

The participants consisted of five diabetes specialists (two trained GPs, two endocrinology subspecialists, and one diabetes nurse) and eight patients who were selected through the convenient sampling method between June and August 2016. Subjects were interviewed for 40 minutes on average. The interviews continued until no new information was added. Table 3 shows the patients’ demographic information.

**Table 2:** Mean scores and standard deviations of health providers’ multiple aspects of content quality (n=3).

Item groups	Evaluation aspects				
	Accuracy	Simplicity	Usefulness	Adequacy	Total
Symptoms	4.9 (0.1)	4.7 (0.2)	4.5 (0)	4.6 (0.2)	4.6 (0.1)
Diseases	4.9 (0.2)	4.6 (0.3)	4.4 (0.1)	4 (0.1)	4.4 (0.3)
Drugs	4.8 (0.1)	4.3 (0.3)	4 (0.1)	4.6 (0.2)	4.4 (0.2)
Tests	5 (0)	4.8 (0.1)	5 (0)	5 (0)	4.9 (0.05)
Total score	4.9 (0.1)	4.6 (0.2)	4.4 (0.05)	4.5 (0.1)	

**Table 3:** Patient participants’ demographic information.

Category	Variable	Value
Demographic	Average age (range), in years	63 (57-68)
	Time since diagnosis (mean), in years	12
	Affliction with consequences, in years	4
Medical diet	Drug	3
	Drug + insulin	5
Education	˂diploma	5
	≥diploma	3
Information sources	Press and TV	5
	Kith and kin	6
	Reading books or educational brochure	4
	Class attendance	4

After the interviews, the audio record was transcribed and then analyzed based on TAM. The analysis involved an identification and categorization of key statements. To do this, each response was analyzed line by line in the related context, and finally the following three themes were found for perceived usefulness: (1) raising patient knowledge and self-awareness; (2) improving patient self-care; and (3) improving doctor-patient interaction.

The specialists’ and patients’ attitudes toward each of the three themes are presented below.

#### Raising Patient Knowledge and Self-Awareness

The most remarkable aspect of the system, according to the specialists, is informing patients and making them aware of their medical condition. In this regard, specialist number 1 states:

one medical goal for diabetic patients is to make them aware of their disease and equip them with self-care knowledge. Experience has shown that active patients who gain information from different sources manage to control their blood sugar better…

The patients also maintained that access to their records and its explanations is like carrying a full-time tutor who can be accessed anytime and anywhere. Patient number 2 mentioned that the key to entering the world of diabetes is familiarity with the language of this disease. He continued:

…To comprehend texts on diabetes, one needs to have a good command of two languages, the language in which the text is written and the language of diabetes….

According to the specialists, providing feedback about patient status based on his or her lab test results along with general explanations not only raises patient awareness of one’s own conditions but also encourages one to read more on the topic. With this concern, patient number 7 stated:

within the 20 years I have been suffering from diabetes, I’ve read many books and materials. Though I already knew much of what was presented in this system, still when I think this content is especially prepared for me, it becomes more interesting and I tend to read more

#### Improving Patient Self-Care

Specialists believed that patient access to his or her record is accompanied by more feelings of responsibility for self-care, which can lead to delayed emergence of complications. In this respect, specialist number 3 maintains:

…In my opinion, information tailoring is a sort of attention paid to patients. The short time of the visit does not let every patient's questions be answered and s/he might feel ignored. This method of addressing an individual creates a feeling of importance in him/her, and further motivates reading and practical application. All this makes one believe in one’s role in actively managing his/her disease…

According to his experience of hypoglycemia, patient number 5 stated:

I did not know then why I was feeling that way, and did not know what I was supposed to do. The explanations provided in the system both involved preventive advice and treatment recommendations that greatly contributed to lowering the frequency and costs of visiting doctors.

#### Improving Doctor-Patient Interaction

Specialists believe that the limited time of a visit will be more effectively used as patient awareness and knowledge improves. One specialist referred back to his own experience of giving instructional leaflets to patients, and believed that patients welcome receiving leaflets from doctors and see it as a sign of respect and attention. He anticipated that patients would read the leaflets, and continued:

…the effect of leaflets on changing a patient’s behavior and performance can be easily seen in the type of questions asked the following session…

Patients also believed that accessing their record and receiving explanations helps them better understand the doctor’s words. A majority of patients have mentioned that they heard at least one of the terms in their records from the doctor. However, they did not know what it meant. With this concern, one of them stated:

having read the record, I came to know that I mispronounced the names of some drugs…

Data concerning perceived ease of use was analyzed in a similar fashion. Patients’ comments were more in the form of suggestions to improve program facilities and did not mention any problems related to interacting with the system. Textbox 1 shows some of the suggestions made by the specialists about the facilities of the system:

The last item in the Textbox 1 implies that some items might be repeated in the patient’s record at different visits. One suggestion was that the information provided should be updated each time. For example, details on blood fat begin with simple basic issues, and once the patient’s knowledge is raised over time, more issues that are complex are offered in the following sessions.

Here are some of the issues extracted from patients’ comments with respect to perceived ease of use.

Access to all library content: Currently, patients can only view that portion of information that is contained within their record. One suggestion is to provide access to all library content in a tree-like menu. This helps the patient to access his or her record content or search for any other relevant issue that might occur later.

Allowing the patient to enter his or her lab test values and receiving their interpretation: One of the most interesting parts of the program for patients is the analysis of their lab test results along with appropriate advices. At present, the program is designed in a way that the patient-user cannot enter data directly into the system, but can just ask for any data already entered by the doctor. The patients made a request for the possibility of entering lab test results and receiving the interpretation before visiting the doctor. Although this was not considered among the system’s primary goals, due to the importance of this feature in better informing the patients and preparing them for the visiting session, it was added to the system as a secondary feature. The mobile-based version of the program, and the possibility of doctor-patient interaction and Q&As were among the other suggestions.

**Suggestions made by specialists about the facilities of the system.**

- Considering a possibility of adding to and updating library content by the doctor-user.
- Visualizing test results for a better understanding of less literate patients.
- Taking advantage of audio-visual facilities for more effectiveness.
- Updating the content provided for the patient.

## Discussion

### Principal Findings

This research dealt with the design, implementation, and evaluation of the first version of a self-describing, tailored, diabetes medical record system. The system design is based on a general approach with no local feature consideration, so it can be adapted to any health care setting with minimum modification, if required. Providing didactic information in the context of medical records is a novel field in information tailoring systems.

According to the body of research, this study is the first step toward providing tailored explanations for diabetic patients in the Persian language. In this research, tailoring is done based on the content of medical records comprising demographic information as well as patients' medical history. Although adding more aspects could improve the quality of information tailoring, there must be a trade-off between the costs of processing more data and raising the quality of the tailored information. Taking records as the basis of identifying relevant topics and interpreting patients’ status avoids the data entry burden on the user.

A great body of research into tailored information provision systems is focused on evaluating system effectiveness on patient behavioral and clinical outcomes. A study [20] was conducted on tailored educational materials based on health literacy level and diabetic patients’ learning style. The study examined the effectiveness of tailored information on promoting the knowledge of 160 diabetic patients using RCT design. The results obtained revealed that the knowledge of patients who had received tailored educational products was significantly higher than the control group. Another investigation was the “Move more for life” system [21] that produced a tailored educational pamphlet for survivors of breast cancer and evaluated its effect on 330 participants. The results showed that subjects in the intervention group did resistance physical exercises three times more than the control group. A weakness of these investigations is their inadequate elaboration on the features and functions of the tailoring system. In our study, precise and detailed system description helped to fill the gap and facilitate the design of similar systems in the future.

Outcome-based evaluation methods require patients’ extensive cooperation and are at risk of unpredicted human and contextual issues, which necessitate considerable time, money, and multiple resources. The researchers' extensive understanding of underlying influential features and the audience’s needs and preferences can lead to a more effective system design. Qualitative research provides the researcher with more access to the inner world of an individual, his or her values, preferences, attitudes, and beliefs. In our previous research we suggested that objective criteria do not necessarily reflect actual user satisfaction and that measuring user perceived satisfaction is a more reliable criterion for judging the system’s effectiveness in practice [22]. Our study setting was undergoing a transition phase from paper to EMR and, thus, the evaluation approach in our study belongs to the qualitative domain and includes field observations and qualitative interviews. One of the strengths of this research is the consideration of the target audience’s attitudes in all three phases of the study: need analysis, design and implementation, and evaluation.

A majority of computer programs are designed based on the system designers' assumptions about the users’ information requirement. This stands as a key reason for the users’ low acceptance [12]. Therefore, the first phase of this research dealt with an examination of the target population information needs following field observations.

The final system evaluation was done using a qualitative approach and semistructured interview. In this research, the attitudes of two groups (doctors and patients) were investigated simultaneously, which can be considered as a strength of this study. The results obtained from field observations and interviews have shown that the audiences were ready to accept the system and they had a positive attitude toward using it in practice.

As generalizability is not the purpose of qualitative evaluation methods, we do not make any claim about the applicability of our findings to a wider population or to different contexts. However, comparing the data in Table 3 with the results obtained in study [23], we can consider the study participants both demographically and situationally representative of diabetic patients in Iran. Therefore, we do not expect a dramatic difference in results when recruiting different participants in different contexts.

A substantial *body of literature* has mentioned the important role of users’ mental acceptance in increasing the probability of success in transition from paper to an EMR [24]. Hence, findings from our study about the users’ readiness and attitudes provide a valuable perspective for health care policy makers in Iran to make more informed decisions in the transfer process.

The unique feature of the system from both the perspectives of patients and specialists is the tailored sense of the information and its effect on motivating one to learn and act better. In this regard, article [22] indicates that the closer the content is to the patient’s needs, wants, and perception level, the better that information is digested by the reader and the more practical it becomes.

A comparison of the usability evaluation of the two groups of patients and doctors indicated that patients tended more toward the superficial aspects of the program, whereas doctors’ comments were more focused on the procedures and facilities of the program. Together, these two can provide a complete view of the system.

Usability testing conducted on the patients revealed that they preferred to receive the information in the print-based format, which is consistent with the findings of the review article [25]. In this research, those who had received the information printed read the content more than those who received the information in digital form. Similarly, according to the review article [26], despite the provision of audio-visual products and Internet-based programs, paper pamphlets continued to be the most prevalent way of communicating information to the patients. The majority of patients preferred to receive content in print format to study as much as needed in a more relaxed context.

### Limitations

The limited number of participants and the limited exposure time to the system are the limitations of this research, which can affect the generalizability of the findings.

The study limitations included incompleteness of some of the patients’ data in paper-based records and doctors’ illegible handwriting. As this research is a pilot study, technical limitations are not considered at this phase. However, the coverage, speed, and accessibility of the Internet network in Iran are a challenge that needs special attention when implementing the system in reality.

### Future Work

Due to the importance of psychological issues in the care of diabetic patients, we suggest considering this aspect in future research in order to achieve more fine-grained tailoring of information. Furthermore, a part of users' suggestions mentioned in the result section can be the basis of future works. Our latter suggestion for future studies is to investigate the long-term effect of the system on patients’ outcomes, such as their increased knowledge and self-awareness compared with the usual information provision method.

## Appendix

Multimedia Appendix 1 Two overviews for patient's data representation.

Multimedia Appendix 2 Evaluation Framework and Interview Guide.

Multimedia Appendix 3 Complete list of questions patients asked in observational study.

Multimedia Appendix 4 Content Quality Assessment.

## Abbreviations

EMR: electronic medical records
GP: general practitioner
TAM: technology acceptance model

## References

[1] Lotfi M, Saadati H, Afzali M (2014). Prevalence of diabetes in people aged ≥ 30 years: the results of screen-ing program of Yazd Province, Iran, in 2012. J Res Health Sci.

[2] Alharbi N, Alsubki N, Jones S, Khunti K, Munro N, de LS (2016). Impact of information technology–based interventions for type 2 diabetes mellitus on glycemic control: a systematic review and meta-analysis. J Med Internet Res.

[3] Schulz P, Nakamoto K (2013). Health literacy and patient empowerment in health communication: the importance of separating conjoined twins. Patient Educ Couns.

[4] Office for Civil Rights‚ HHS (2001). Standards for privacy of individually identifiable health information. Final rule; correction of effective and compliance dates. Fed Regist.

[5] Ross S, Lin C (2003). The effects of promoting patient access to medical records: a review. J Am Med Inform Assoc.

[6] Davis GT, Menon S, Parrish D, Sittig D, Singh H (2014). Patient access to medical records and healthcare outcomes: a systematic review. J Am Med Inform Assoc.

[7] Kreuter MW (2000). Tailoring health messages: customizing communication with computer technology.

[8] Froschl C (2005). IICM.

[9] Harrington NG, Noar SM (2012). Reporting standards for studies of tailored interventions. Health Educ Res.

[10] McRoy SW, Liu-Perez A, Ali SS (1998). Interactive computerized health care education. J Am Med Inform Assoc.

[11] DiMarco C, Hirst G, Wanner L, Wilkinson J (1995). HealthDoc: customizing patient information and health education by medical condition and personal characteristics.

[12] Buchanan BG, Moore JD, Forsythe DE, Carenini G, Ohlsson S, Banks G (1995). An intelligent interactive system for delivering individualized information to patients. Artif Intell Med.

[13] Hartson R (2012). The UX Book: Process and Guidelines for Ensuring a Quality User Experience.

[14] Van Velsen L, Van Der Geest T, Klaassen R, Steehouder M (2008). User-centered evaluation of adaptive and adaptable systems: a literature review. Knowl Eng Rev.

[15] Delone W (2003). The DeLone and McLean model of information systems success: a ten-year update. JMIS.

[16] Lee Y, Kozar K, Larsen KR (2003). The technology acceptance model: past, present, and future. CAIS.

[17] Lee K, Hoti K, Hughes J, Emmerton L (2015). Consumer use of “Dr Google”: a survey on health information-seeking behaviors and navigational needs. J Med Internet Res.

[18] Davis FD (1989). Pusefulness, perceived ease of use, and user acceptance of information technology. MIS Quarterly.

[19] Brooke J (1996). Meiert.

[20] Koonce T, Giuse N, Kusnoor S, Hurley S, Ye F (2015). A personalized approach to deliver health care information to diabetic patients in community care clinics*†. J Med Libr Assoc.

[21] Short C (2013). Theory-and evidence-based development and process evaluation of the Move More for Life program: a tailored-print intervention designed to promote physical activity among post-treatment breast cancer survivors. Int J Behav Nutr Phys Act.

[22] Tara M (2015). IRMA.

[23] Veghari G, Sedaghat M, Joshaghani H, Hoseini SA, Niknezad F, Angizeh A, Tazik E, Moharloei P (2010). Association between socio-demographic factors and diabetes mellitus in the north of Iran: a population-based study. Int J Diabetes Mellit.

[24] Abdekhoda M, Ahmadi M, Dehnad A, Hosseini A (2014). Information technology acceptance in health information management. Methods Inf Med.

[25] Pal K, Eastwood S, Michie S, Farmer A, Barnard M, Peacock R, Wood B, Edwards P, Murray E (2014). Computer-based interventions to improve self-management in adults with type 2 diabetes: a systematic review and meta-analysis. Diabetes Care.

[26] Kenny T, Wilson R, Purves I, Clark JS, Newton L, Newton D, Moseley D (1998). A PIL for every ill? Patient information leaflets (PILs): a review of past, present and future use. Fam Pract.

