# Supplementary material for: Design, Implementation, and Evaluation of Self-Describing Diabetes Medical Records: A Pilot Study
Source: JMIR Med Inform. 2017 May 2;5(2):e10. doi: 10.2196/medinform.6862 (PMC5434252; doi:10.2196/medinform.6862)
Supplement: Multimedia Appendix 1 [file medinform_v5i2e10_app1.pdf]

Multimedia Appendix 1: Two overviews for patient's data representation

Session-based View

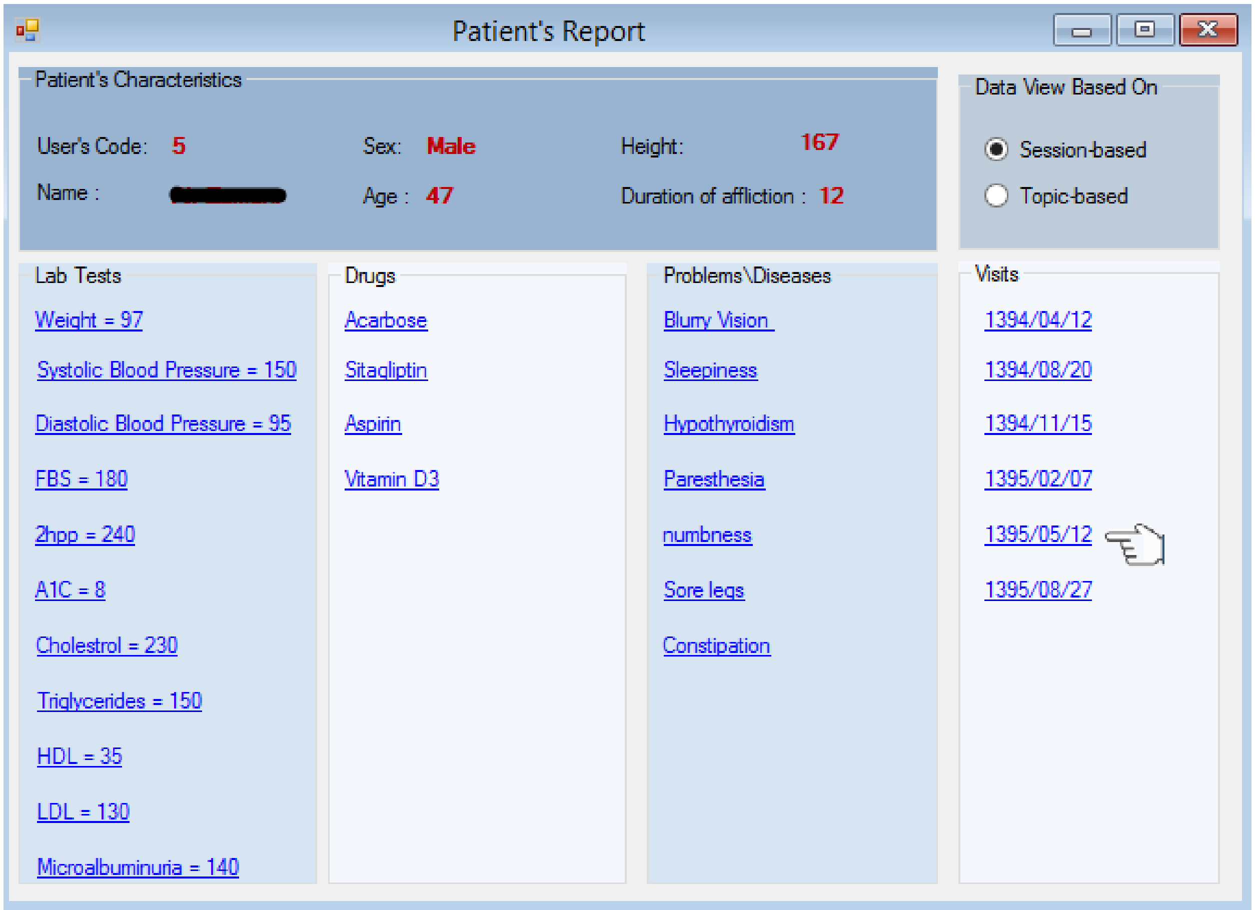

Topic-based View

This data view mode provides a visual abstract overview of the chronological progress for the selected data elements in patient’s record.

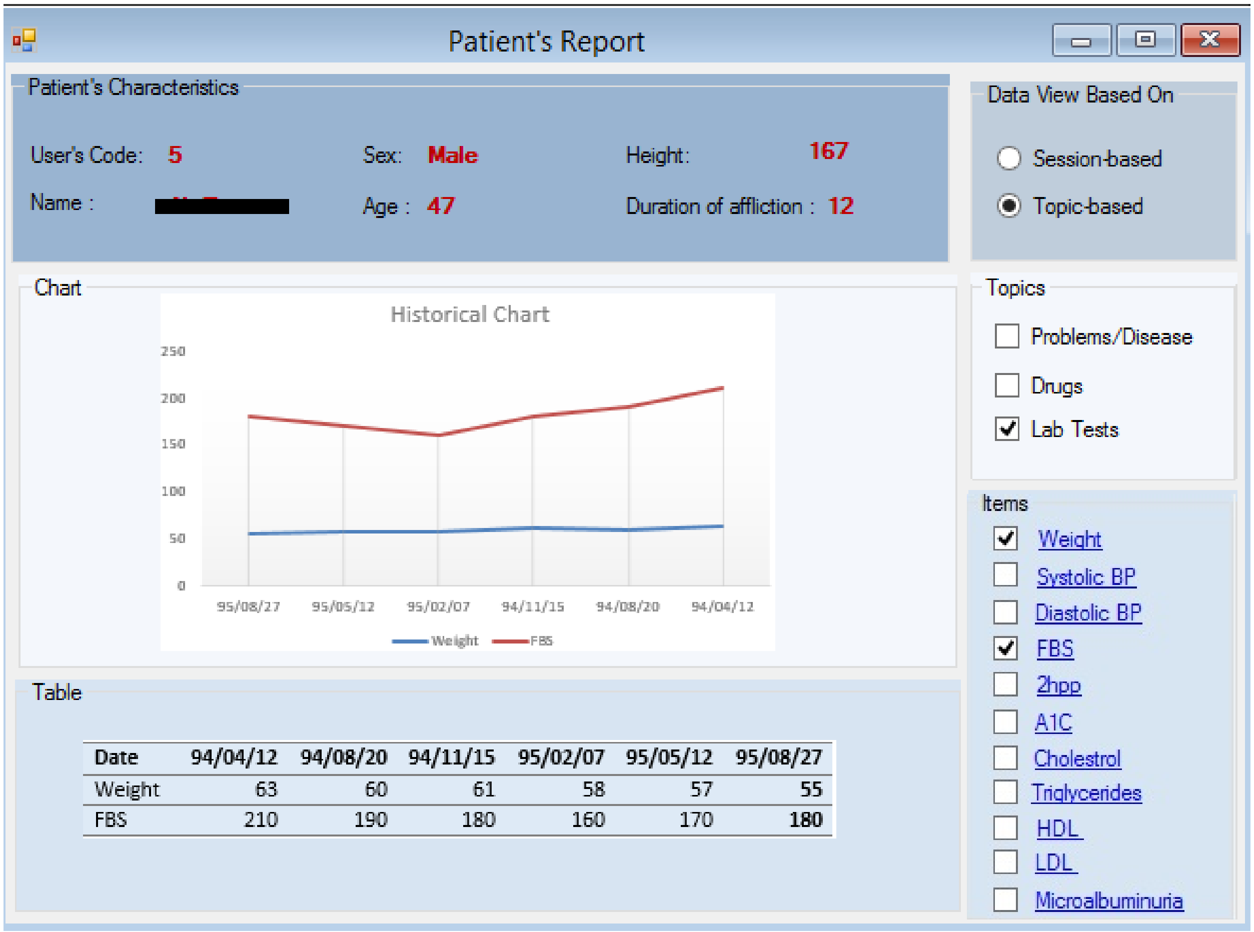

As indicated in the figure, in this view mode, a patient can see his/her recorded information ever since the first visit, thematically divided. Here, the information is displayed based on the selected topic category ("Topic" box in the figure). Accordingly patient can see list of all items in his/her record related to that topic ("Items" box in the figure). Selecting any of the items leads the patient to a display of the temporal trend of the topic both in graphical and tabular format ("Chart" and "Table" boxes in the figure). Moreover, due to space limit and avoidance of information overload, the user can select maximally 3 items at a time and view the comparative trend of change. Following each item's selection or deselecting by the user, the graphical display will be dynamically updated.

Similar to the Session-based display mode, as can be seen in the figure, the item list is in the form of a hypertext as a click on each item the relevant explanation box will appear. The figure above shows a patient's lab items as the selection of the two parameters weight and FBS indicates the trend of change from the beginning so far. This mode of abstract display provides users with the chance of assessing their performance over time and can influence on their decisions for future self-care plans.
