# Supplementary material for: Design, Implementation, and Evaluation of Self-Describing Diabetes Medical Records: A Pilot Study
Source: JMIR Med Inform. 2017 May 2;5(2):e10. doi: 10.2196/medinform.6862 (PMC5434252; doi:10.2196/medinform.6862)
Supplement: Multimedia Appendix 2 [file medinform_v5i2e10_app2.pdf]

Multimedia Appendix 2: Evaluation Framework and Interview Guide

Evaluation Strategy

We developed a two-stage evaluation framework, presenting an overall view of the main aspects of measurement for the present study. A validated questionnaire with 20 items (10 dealt with the perceived usefulness and 10 dealt with perceived ease of use), developed by Davis (1989), formed the basis for designing the interview questions. This guide consists of two parts: assessing perceived usefulness and assessing perceived ease of use.

Part One: Perceived usefulness

According to Davis (1989) Perceived usefulness is defined as "the degree to which a person believes that using a particular system would enhance his or her job performance."
This construct is measured through a semi-structured interview. The underlying model to develop the interview questions is comprised of three dimensions: scales, domains, and functions. A detailed description for this triangle framework are provided below.

1st dimension: assessment scales

The following 8 indices were taken from the Davis's validated questionnaire (Davis 1989) for perceived usefulness. The definition of each scale is provided in the reference (Davis 1989). items fall into three main clusters. The first cluster relates to job effectiveness, the second to productivity and time savings, and the third to the importance of the system to one's job.

- Job Effectiveness
  - Quality of work
  - Job performance
  - Effectiveness on the job
- Productivity And Time Savings
  - Work More Quickly
  - Increase Productivity
  - Accomplish more work
- The Importance Of The System To One's Job
  - Control over work
  - Make job easier

2nd dimension: assessment domains

The general terms 'work' and 'job' used in the definition of scales were replaced with the following phrases in our study:

- perception and access to the required data
- doctor-patient interaction

- intention/ability to do self-care activities

3rd dimension: Question words

Each scale in each domain can be enquired in three forms:

- 'WHAT' questions: Assess user's perception of the role and the effects the system can provide to improve works.
- 'HOW' questions: Assess user's perception of the quality and the extent the system can improve works.
- 'IN WHAT WAY' questions: Assess user's perception of ways the system can affect and improve works.

So far, our framework contains 8 scales, 3 are domains and 3 types of question words, which makes it possible to have an overall 72 independent questions measuring perceived usefulness (Each scale can be questioned in 3 domains and 3 types (8*3*3)). The following table elaborates on the three-dimensional structure of the framework.

**Q. Type**<br><br>**Aspect**	**What role do you conceive for the system in**	**In your opinion, How can the system**	**In what ways can the use of this system**
**Understand/ Access Information**	.Using this product improves the quality of the work I do		
	.Using this product gives me greater control over my work		
	.This product enables me to accomplish tasks more quickly		
**Doctor-Patient Interaction**	.This product increases my productivity		
	.This product improves my job performance		
	This product allows me to accomplish more work than would otherwise be .possible		
**Ability to Do Self-care Activities**	.This product enhances my effectiveness on the job		
	.This product makes it easier to do my job		

A juxtaposition of the values in each row, column and cell forms one unique question. For instance, in the case of 'Understand Information', in the 1st dimension, a 'what' question from the 2nd dimension, the quality of work from the 3rd dimension, the following question will be developed:

"What role do you conceive for the system in promoting/ increasing patient's knowledge and self-awareness about the disease?"

Part Two: Perceived ease of use

According to Davis (1989) Perceived ease of use is defined as "the degree to which a person believes that using a particular system would be free of effort.". The following measurement scales are introduced by Davis for this constitute:

- Physical Effort
  - Cumbersome
  - Controllable

    - Rigid & Inflexible
- Mental Effort
    - Frustrating
    - Mental Effort
    - Understandable
- Easy To Learn
    - Ease of Learning
    - Ease of Remembering
    - Effort to Be Skillful

As stated in the main body of the article, perceived ease of use is measured in two phases:

- Initially, after providing the required explanations about how to work with the program, the user was asked to utter out whatever s/he felt and thought about interacting with the system while doing the task of entering the record information. Meanwhile, the researcher allowed the user to act spontaneously with no disturbance or questioning.
- Finally at the closure of the session, selected items from standardized SUS, and Davis(1989) questionnaires, as shown in the following table, were asked.

The data collection method throughout the research was voice recording and note-taking.
As revealed by the definition, a semi-structured interview is not restricted to certain set of questions or choices and the interviewee is free to answer in an open-ended manner. So, keeping the main track, the interviewer can ask further questions besides those included in the predefined list. Consequently, not all the questions mentioned here were necessarily asked in each interviews session.

**No.**	**Question (Questionnaire)**
1	How frequently would you like to use this system? (SUS)
2	Do you think the system was easy to use? (SUS)
3	Do you think you need the support of a technical person to be able to use this system? (SUS)
4	Do you feel confident using the system? (SUS)
5	How easy was the Learning to operate the system? (PUEU)
6	Did you find it easy to get the system to do what you want it to do? (PUEU)
7	Was your interaction with the system clear and understandable? (PUEU)
8	Do you think it would be easy for you to become skillful at using the system? (PUEU)

References:

Davis FD. Perceived usefulness, perceived ease of use, and user acceptance of information technology. MIS quarterly. 1989 Sep 1:319-40.

Davis FD. User acceptance of information technology: system characteristics, user perceptions and behavioral impacts. International journal of man-machine studies. 1993 Mar 31;38(3):475-87.
