# Supplementary material for: Design, Implementation, and Evaluation of Self-Describing Diabetes Medical Records: A Pilot Study
Source: JMIR Med Inform. 2017 May 2;5(2):e10. doi: 10.2196/medinform.6862 (PMC5434252; doi:10.2196/medinform.6862)
Supplement: Multimedia Appendix 3 [file medinform_v5i2e10_app3.pdf]

Multimedia Appendix 3: Complete list of questions patients asked in observational study (visits sessions and educational classes)

Row	category	Concept
1	symptoms	1. enquiry about the cause of disease<br>2. enquiry about how to manage or control the symptoms and side effects
2	Oral Drugs	1. indications (administration and dosage)<br>2. medical interactions<br>3. how to make up for the forgotten dosage<br>4. side effects<br>5. herbal medications<br>6. advertised products (in mass media)<br>7. manufacturer<br>8. costs
3	Insulin	1. how to inject<br>2. how to use the flex pen<br>3. injection time schedule<br>4. mechanism of action of insulin within body<br>5. combined effect with oral drugs<br>6. costs
4	Tests	1. definition of the abbreviations used in test result<br>2. one's status in terms of each and every test parameter<br>3. possible reasons for abnormal test results<br>4. request for recommendations or instructions for maintaining the parameter within the normal range<br>5. required conditions for having the test<br>6. costs of the test<br>7. enquiry about recommending a reliable lab
5	Blood Glucose Test	1. how to use the glucometer<br>2. time and frequency of measures<br>3. how to interpret the results<br>4. costs
6	Nutrition	1. permitted load of food materials<br>2. type of food stuff appropriate for each meal<br>3. meal intervals<br>4. effect of certain foods on blood glucose, pressure, etc.<br>5. weight loss/gain
7	Physical Activity	1. type of activity fitted to one's conditions and movement limitations<br>2. time needed for physical activity<br>3. intensity of the activity<br>4. pre- and post-activity care and considerations<br>5. problems occurring at or prior to the activity
8	Psychological Problems	1. fatigue and lethargy<br>2. depression<br>3. feeling guilty due to costs imposed on family and healthcare

		4. anxiety in emergency conditions e.g. pressure drop
9	Exposure to New Circumstances	1. special care and simultaneous management of multiple diseases<br>2. effect of new disease on the current therapy<br>3. how to change the present therapy to meet new conditions<br>4. asking doctor to recommend a reliable medical center for constant visits and following complications and other diseases<br>5. occasional questions: trips, fasting, pregnancy, etc.
