# Supplementary material for: Design, Implementation, and Evaluation of Self-Describing Diabetes Medical Records: A Pilot Study
Source: JMIR Med Inform. 2017 May 2;5(2):e10. doi: 10.2196/medinform.6862 (PMC5434252; doi:10.2196/medinform.6862)
Supplement: Multimedia Appendix 4 [file medinform_v5i2e10_app4.pdf]

Multimedia Appendix 4: Content Quality Assessment

To assess the quality of the collected information, a booklet was availed to three experts (one endocrinology sub-specialist, one GP who had passed a diabetes course, and one nurse who was a diabetes educator), which was comprised of four sections (symptoms, diseases, drugs and tests). There were 110 pieces of information (each elaborating on one item which was probably present in diabetic patients' record). Expert assessment was done along 4 axes: accuracy, simplicity, usefulness and adequacy. Tables 1-3 below indicate the 3 experts' assessment respectively. Table 4 shows the mean rating score of tables 1 to 3 for each index. And, table 5 included in the main manuscript also presents these scores in percentages.

Table1. Endocrinology Sub-specialist

Evaluation aspect / Item group	Accuracy	Simplicity	Usefulness	Adequacy	Total
Symptoms	5	5	4.5	5	4.8
Diseases	5	5	4.5	4.2	4.8
Drugs	5	4.5	4.2	5	4.8
Tests	5	5	5	5	5
Total score	5	4.8	4.5	4.8	*

Table2. Trained GP

Evaluation aspect / Item group	Accuracy	Simplicity	Usefulness	Adequacy	Total
Symptoms	5	4.5	4.5	4.5	4.6
Diseases	5	4.5	4.5	4	4.5
Drugs	4.7	4.6	4	4.5	4.4
Tests	5	4.7	5	5	4.9
Total score	4.9	4.5	4.5	4.5	*

Table3. Diabetes Educator (nurse)

Evaluation aspect / Item group	Accuracy	Simplicity	Usefulness	Adequacy	Total
Symptoms	4.7	4.75	4.5	4.5	4.6
Diseases	4.6	4.3	4.2	4	4.2
Drugs	4.7	4	4	4.5	4.3
Tests	5	4.7	5	5	4.9
Total score	4.7	4.4	4.4	4.5	*

Table 4. Mean (SD)

Evaluation aspect / Item group	Accuracy	Simplicity	Usefulness	Adequacy	Total
Symptoms	4.9 (0.17)	4.7(0.25)	4.5(0)	4.6 (0.28)	4.6 (0.11)
Diseases	4.9 (0.23)	4.6(0.36)	4.4 (0.17)	4 (0.11)	4.4 (0.3)
Drugs	4.8 (0.17)	4.3(0.32)	4 (0.11)	4.6 (0.28)	4.4 (0.26)
Tests	5 (0)	4.8 (0.17)	5 (0)	5 (0)	4.9 (0.05)
Total score	4.9 (0.15)	4.6(0.20)	4.4 (0.05)	4.5 (0.17)	*

Mean scores were reported in percentage through the following relation (table 5).

X= (Mean*100)/5

Table 5. Health providers' rating of multiple aspects of content quality

Evaluation aspect / Item group	Accuracy	Simplicity	Usefulness	Adequacy	Total
Symptoms	98.3%	95%	90.0%	93.3%	94.1%
Diseases	97.2%	92%	88.6%	82%	90%
Drugs	96.2%	87.4%	82.1%	93.3%	89.7%
Tests	100%	96.3%	100%	100%	99%
Total score	97.9%	92.6%	90.1%	92.1%	*

Here we provide a brief interpretation for table 2's data in two dimensions: column-based and row-based.

Column-based interpretation:

Criteria for assessing the quality of content comprise: accuracy, simplicity, usefulness, and adequacy (table's four columns). These criteria can be categorized into two aspects. Intrinsic aspect (include accuracy) which evaluates the information quality itself, regardless of the context. The assessment is based on the expert's opinion (expertise knowledge). All three evaluators assigned the highest score to this scale, which shows high reliability on information correctness.

On the other side we have contextual aspects (simplicity, usefulness, and adequacy) which consider the effects of other factors when judging the quality of the information.

For contextual aspects we asked experts' perception about the information simplicity, usefulness, and adequacy regarding the patient's level of knowledge and ability. The closer expert's assumptions about patients' needs and abilities to the reality, the more precise and reliable the assessment will be. As can be seen in the table the total score for usefulness is the lowest particularly for drug category, which can be due to the highly specialized nature of this domain with difficult and diverse drug's generic and brand names. We improved the information in this section based on expert's comments. Scores in simplicity and adequacy criteria were highly satisfactory and nearly the same for all topic categories.

Row-based interpretation:

Looking to the total scores in row dimension reveals that information about lab tests achieved the highest scores in each criteria which shows its importance and approval by the experts.

Scores on the other three categories (Symptoms, Disease, Drugs) were nearly the same but the symptoms was slightly better.
